# Placental metabolic profiling in gestational diabetes mellitus: An important role of fatty acids

**DOI:** 10.1002/jcla.24096

**Published:** 2021-11-09

**Authors:** Yuqi Yang, Zhaoping Pan, Fang Guo, Huihui Wang, Wei Long, Huiyan Wang, Bin Yu

**Affiliations:** ^1^ Department of Medical Genetics Changzhou Maternal and Child Health Care Hospital affiliated with Nanjing Medical University Changzhou China; ^2^ Department of Obstetrics Changzhou Maternal and Child Health Care Hospital affiliated with Nanjing Medical University Changzhou China

**Keywords:** fatty acids, gestational diabetes mellitus, mass spectrometry, metabolic profiling, placenta

## Abstract

**Aim:**

Gestational diabetes mellitus (GDM) is the most common metabolic disorder during pregnancy. Accumulating studies have reported metabolites that are significantly associated with the development of GDM. However, studies on the metabolism of placenta, the most important organ of maternal‐fetal energy and material transport, are extremely rare. This study aimed to identify and discuss the relationship between differentially expressed metabolites (DEM) and clinical parameters of the mothers and newborns.

**Methods:**

In this study, metabolites from 63 placenta tissues (32 GDM and 31 normal controls) were assayed by ultra‐performance liquid chromatography‐high resolution mass spectrometry (UPLC‐HRMS).

**Results:**

A total of 1297 annotated metabolites were detected, of which 87 significantly different in GDM placenta. Lipids and lipid‐like molecules accounted for 62.1% of DEM as they were significantly enriched via the “biosynthesis of unsaturated fatty acids” and “fatty acid biosynthesis” pathways. Linoleic acid and α‐linolenic acid appeared to be good biomarkers for the prediction and diagnosis of GDM. In addition, the level of PC(14:0/18:0) was negatively correlated with neonatal weight. 14 metabolites significantly different in male and female offspring, with the most increase in female newborns.

**Conclusion:**

Even if maternal blood glucose level is well controlled, there are still metabolic abnormalities in GDM. Lipids and lipid‐like molecules were the main differential metabolites, especially unsaturated fatty acids.

## Graphical abstract

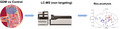

## 1 INTRODUCTION

Pregnancy is accompanied by several metabolic changes which become more intense when women experience complications. Gestational diabetes mellitus (GDM) is the most common metabolic disorder during pregnancy. Although the molecular mechanism of GDM is still unclear, it is well known that the disease is caused by the complex interaction of genetic and environmental factors, accompanied by a series of metabolic changes.^1^, ^2^ Recent advances in metabolomics can provide the most integrated profile of biological status and contribute to better understanding of the etiology and pathogenesis of diseases. GDM has already become one of the subjects of focus in metabonomics with a growing number of studies revealing a range of metabolites, especially including amino acids, organic acids, lipids, and fatty acids, that are significantly associated with the development of GDM.^3^, ^4^

The following types of metabonomics studies on GDM are typical: (1) the most common are investigations for predictive biomarkers which can be used for diagnosis and early risk stratification. Most of these involve studies on blood or urine, as biological specimens. Although many metabolites have been reported, the results are neither consistent nor have they been proven through large sample prospective studies. (2) Exploration of the unique metabolic transition from pregnancy to postpartum. The metabonomic characteristics of the transition from GDM to type 2 diabetes mellitus area recent focus. Allalou^5^ identified 21 metabolites that significantly differed when GDM progressed to type 2 diabetes mellitus. The discriminative power was 83.0%, which was far superior to measuring fasting plasma glucose levels alone. The lipid species CE 20:4, PE(P‐36:2), and PS 38:4 were also reported as significant risk factors for the progression from GDM to type 2 diabetes.^6^ (3) Evaluation of the effect of GDM treatment. Pinto et al.,^7^ reported that the treatment duration of GDM was related to the urine metabolic profile, when they compared the effects of the excreted metabolome of pregnant GDM women after diet and insulin treatments. (4) Metabolic pathogenesis of GDM. Some dysregulated metabolic pathways were proven to be associated with GDM, such as the metabolism of lipids, amino acids, carbohydrates, and purines. Collectively, these metabolomic‐based studies provide new insights into the pathogenesis and potential targets for the treatment and prevention of GDM, mainly through the discovery of useful biomarkers with high sensitivity and specificity.

However, while being the most important organ of maternal‐fetal energy and material transport, studies of metabolism within the placenta are extremely rare. Some reports have suggested that the placenta has unique metabonomic characteristics in pregnancy complications, including preeclampsia,^8^, ^9^ maternal obesity,^10^ spontaneous preterm birth,^11^ and fetal growth restriction.^12^ These studies suggested that metabolic signatures in placentas might reflect changes in the placental microenvironment, which may affect the development of maternal complications, as well as neonatal and/or adult diseases. Despite being the most common pregnancy‐related metabolic disease, studies on the placental metabonomics of GDM are still lacking. Few have reported the metabolomics of GDM placenta. Uhl O et al^13^ demonstrated that the values of dihomo‐gamma‐linolenic acid decreased, while arachidonic acid and docosahexaenoic acid increased in GDM placenta. PC (16:0/20:4) was considered a major source of arachidonic acid in the fetus. Hence, the study of GDM placental metabolome may reveal the essence of abnormal metabolism at the maternal‐fetal interface of GDM.

This study aimed to obtain the comprehensive metabolite profile of GDM placenta through non‐targeted metabonomics, plus elucidate their specific metabolic pathways through bioinformatics analysis. The relationship between differentially expressed metabolites (DEM) and clinical data were analyzed comprehensively. We hope these results will contribute to reveal a new molecular mechanism of GDM.

## 2 MATERIALS AND METHODS

### 2.1 Ethics approval and consent to participate

The study design and protocol were reviewed and approved by the ethics committee of Changzhou Maternal and Child Health Care Hospital affiliated with Nanjing Medical University. Consent has been obtained from each patient or subject, after full explanation of the purpose and nature of all procedures used.

### 2.2 Clinical subjects

A total of 63 pregnant women took part in the study, including 32 with GDM and 31 with normal pregnancies. All of the GDM women were diagnosed according to “Guideline No. 393‐Diabetes in Pregnancy^14^” The normal pregnant women had neither pregnancy complications nor other basic diseases. Their demographic and clinical assessments are presented in Table 1

**TABLE 1:** Demographics and clinical assessments of the subjects

Index	GDM	Control	*p*
Number	32	31	
Maternal			
Age (years)	31.88 ± 5.28	30.81 ± 3.79	0.361
BMI (kg/m^2^)	28.34 ± 3.12	28.14 ± 3.98	0.827
Gestational age of delivery (weeks)	38.75 ± 0.78	38.71 ± 0.63	0.836
Nature conceived	Y	Y	
Singleton pregnancy	Y	Y	
Primigravid	Y	Y	
Ethnicity	Han	Han	
Cesarean section	12.5%	6.25%	
Newborn			
Male	17 (53.12%)	19 (61.29%)	0.513
Female	15 (46.88%)	12 (38.71%)	
Birth weight (g)	3503.75 ± 491.06	3346.45 ± 341.67	0.146

Abbreviations: BMI, Body mass index; GDM, Gestational diabetes mellitus.

### 2.3 Sample collection

Small pieces of placenta tissue (about 2–5 cm; 1.5 cm^3^) were harvested by the umbilical cord insertion, immediately (within 5 min) after birth. A total of 20 mg of placental tissue was homogenized, added to tryptophan‐d5 and palmitic acid‐[13C] 12 as internal standard (1:1, v/v).

#### 2.3.1 Ultra‐performance liquid chromatography separation

Ultra‐performance liquid chromatography‐high resolution mass spectrometry (UPLC‐HRMS) was used in this study. The samples were analyzed with the combined system of Ultimate TM 3000 ultra‐performance liquid chromatography and Q Exactive quadrupole‐Orbitrap (Thermo Scientific,).

The positive ion detection mode metabolites were separated on an Acquity TM HSS C18 column (Waters Co., 1.7 μm, 2.1 × 100 mm) with 0.1% formic acid aqueous solution and acetonitrile as eluents in linear gradient elution mode. The mobile phases in positive ion detection mode were water, acetonitrile, and methanol, and 5 mM ammonium bicarbonate buffer was added to all solvents, and the metabolites were separated on Acquity TM BEH C18 column (WatersCo., 1.7 μm, 2.1 × 100 mm) with the following elution gradient: The organic phase increased from 2% to 100% in 10 min, and the extra 5 min was used for flushing and balancing the column. The flow rate, sample volume, and column temperature of the two methods were set as 0.4 ml/min, 5 μl, and 50℃, respectively.

#### 2.3.2 Detection parameters

Heating electrospray ionization was used in the two detection modes, and the other mass spectrum parameters were the same except for the ionization voltage, which was 4KV in the positive ion detection mode and 3.5KV in the negative ion detection mode. Other mass spectrum parameters were as follows: The sheath gas flow rate is 45 arb, the auxiliary gas flow rate is 10 arb, and the heater temperature is 355℃, the capillary temperature is 320℃, and the ion transmission lens RF level is 55%. Metabolites were detected in full scan mode, with a resolution of 70,000 FWHM, an AGC of 1E6, and a maximum injection time of 200 ms. The resolution of the secondary mass spectrum for data acquisition was 17,500 FWHM.

The raw data acquired by LC‐MS were analyzed by QI software (Waters Corporation), and metabolites were identified based on public databases (http://www.hmdb.ca/; http://www.lipidmaps.org/ and self‐built databases). Principle component analysis (PCA) and (orthogonal) partial least‐squares‐discriminant analysis (O)PLS‐DA were carried out to visualize the metabolic alterations. The metabolites with variable influence on projection (VIP) values larger than 1.0, and *p* values less than 0.05 were considered as differential metabolites.

### 2.4 Statistical analysis

The group differences in maternal age, BMI, gestational age of delivery, newborns’ birth weight, and placental metabolomes were analyzed with Student's t test to compare normally distributed data and with Mann‐Whitney test to compare non‐normal distributions. Chi‐square test was used to compare the distribution of newborns’ gender between the groups. Since it is not known if metabolites can actually predict GDM, we plotted a receiver operating characteristic curve (ROC), and the overall diagnostic accuracy of the test was summarized by area under the ROC curve (AUC). The strength of the linear relationship between metabolites and birth weight of neonates was tested by Spearman rank‐based correlation coefficient. All analyses were performed by IBM SPSS Statistics 26.

## 3 RESULTS

### 3.1 Characteristic of GDM placental metabolome

**FIGURE 1:**
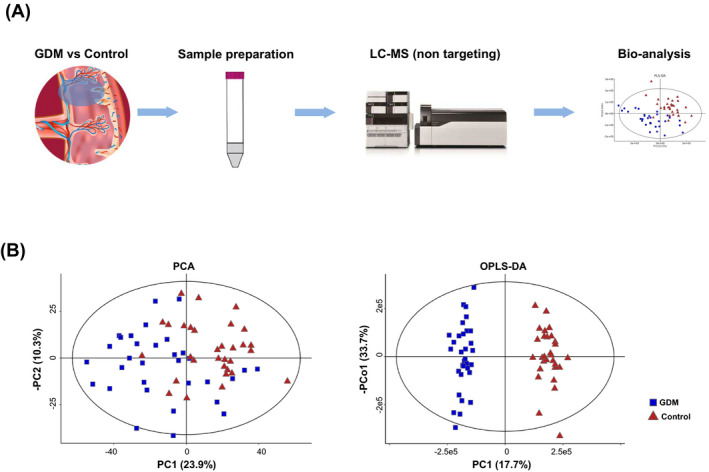
Process of placental metabolic study of GDM. (A) Flow chart of overall research design. (B) Results of PCA and OPLS‐DA analysis

**FIGURE 2:**
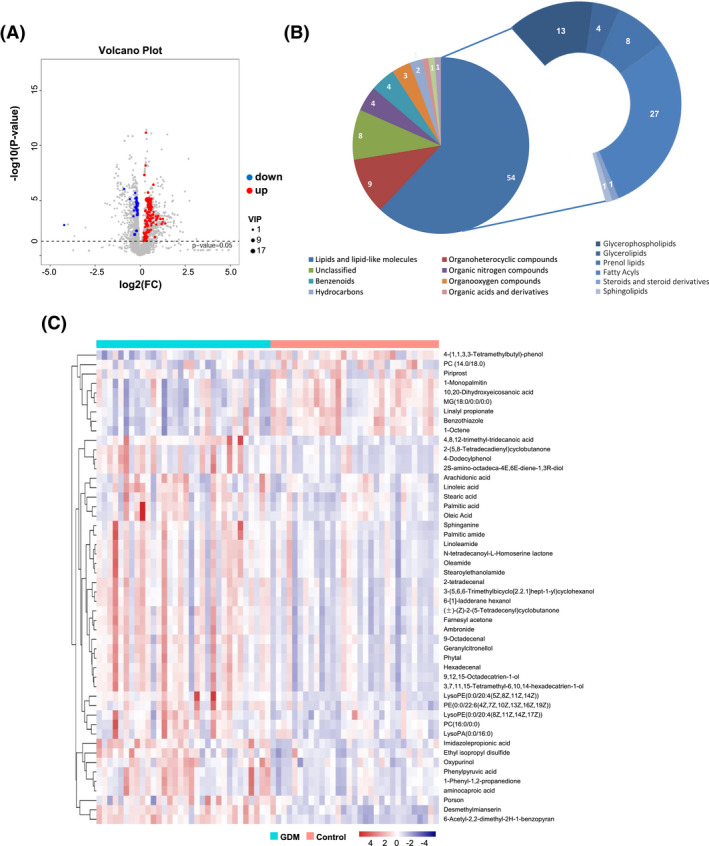
Differentially expressed metabolites (DEM) significantly changed in GDM placenta, (A) Volcano plot of DEM. (B) Classification of DEM. (C) Heatmap of the abundance of top 50 DEM in each sample

Placental metabolites were determined by UPLC‐HRMS, and data were analyzed by PCA and (O)PLS‐DA (Figure 1A). OPLS‐DA score plots showed that two groups (GDM vs control) could be clearly separated (Figure 1B). In total, 1297 annotated metabolites were detected, of which 87 significantly different in GDM placenta, and were thus considered as DEM(VIP>1.0 and *p* < 0.05) (Table S1). Their volcano plot is presented in Figure 2A, wherein 72 increased and 15 decreased. Figure 2B showed the classification of these DEM. The predominant DEMs were lipids and lipid‐like molecules, accounting for 54/87 types (62.1%). Of which, fatty acyls, glycerophospholipids, and prenol lipids were the major three subtypes, comprising 50.0%, 24.1%, and 14.8%, respectively. After hierarchical clustering, a heatmap (Figure 2C) showed relative abundance of the top 50 DEM in each sample, which clearly displayed the significant separation between GDM cases and control.

**FIGURE 3:**
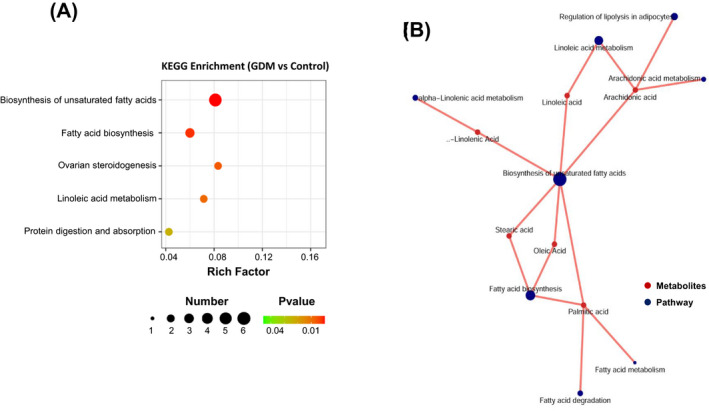
KEGG pathway maps. (A) The top 5 statistically significant pathways in GDM. (B) Relationship between metabolites and pathways

Based on the DEM, pathway enrichment was performed by KEGG pathway maps. The top 5 statistically significant pathways of the DEM are presented in Figure 3A. Notably, the DEM in GDM placenta was significantly enriched to “biosynthesis of unsaturated fatty acids” and “fatty acid biosynthesis” pathways, involving 6 lipid molecules (arachidonic acid, palmitic acid, oleic acid, stearic acid, linoleic acid, α‐linolenic acid). Compared to that of normal pregnant women, these six types of fatty acids significantly increased in the placenta of GDM women (*p* < 0.05). The relationship between metabolites and pathways was then analyzed with R pack (3.6.2) (Figure 3B), and it clearly demonstrated how the fatty acids were involved in the metabolic pathways.

### 3.2 Clinical evaluation and analysis

**FIGURE 4:**
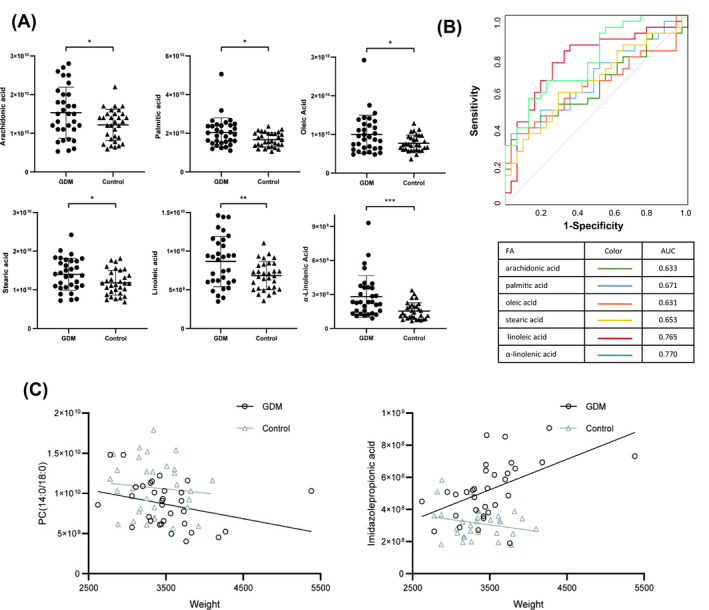
Comparison of fatty acid levels (GDM vs Control). (A) Six types of fatty acids in placenta. (B) ROC curve of six fatty acids. (C) The correlation between the different metabolites and newborn birth weight. Note: *p < 0.05; **p < 0.01; ***p < 0.001

We compared the abundance of the six fatty acids in placental tissues. The levels of arachidonic acid, palmitic acid, oleic acid, stearic acid, linoleic acid, and α‐linolenic acid significantly increased in GDM placenta compared with those of normal pregnant women (*p* < 0.05) (Figure 4A). We then evaluated whether these six fatty acids could also predict the occurrence of GDM. ROC curve analysis confirmed the association. The areas under the curve, 95% confidence interval, and p value are listed in Figure 4B. AUC of linoleic and α‐linolenic acid were higher than 0.75 (Figure 4B).

Secondly, we analyzed the correlation between the different metabolites and some clinical parameters of mothers and newborns. Table 2 showed the statistically significant results. There were many correlations between the DEM and maternal age, gestational age of delivery, newborn birth weight. Due to timely prenatal intervention of GDM women, the birth weight of newborns increased slightly, although it was not statistical significant. The incidence of macrosomia also did not significantly increase. However, it is still worth noting that there were four metabolites related to the newborns birth weight. For example, the level of PC(14:0/18:0) was negatively correlated with neonatal weight. Strangely, there was a positive correlation between imidazole propionic acid and birth weight in GDM, and conversely a negative correlation in normal pregnant women (Figure 4C). Whether these can be used as a predictor for assessing the birth weight still needs to be further explored. Surprisingly, there was no statistically significant correlation between all of the DEM and maternal BMI or blood glucose level.

**TABLE 2:** Results of correlation analysis between metabolites and clinical parameters (*p* < 0.05)

Metabolites	Super Class	UPLC‐HRMS			Correlation	
		VIP	*p* value	FC	r	*p* value
Newborn birth weight						
PC(14:0/18:0)	Lipids and lipid‐like molecules	5.12	0.01	0.81	−0.4128	0.0189
Imidazolepropionic acid	Organoheterocyclic compounds	2.27	<0.0001	1.66	0.5530	<0.000110
6‐Acetyl−2,2‐dimethyl−2H−1‐benzopyran	Organoheterocyclic compounds	2.01	<0.0001	1.25	0.4933	<0.000141
Armillarin	Lipids and lipid‐like molecules	1.05	0.04	1.14	−0.4018	0.0227
Maternal age						
Oleic Acid	Lipids and lipid‐like molecules	5.20	0.02	1.30	−0.3750	0.0344
Linoleic acid	Lipids and lipid‐like molecules	5.07	0.01	1.27	−0.4331	0.0133
MG(18:0/0:0/0:0)	Lipids and lipid‐like molecules	3.62	<0.0001	0.87	0.3697	0.0373
Ethyl isopropyl disulfide	Organosulfur compounds	3.35	<0.0001	1.21	−0.4103	0.0197
1‐Phenyl−1,2‐propanedione	Benzenoids	2.38	<0.0001	1.37	−0.4434	0.0110
Aminocaproic acid	Lipids and lipid‐like molecules	2.25	<0.0001	1.32	−0.4199	0.0167
Phenylpyruvic acid	Benzenoids	2.04	<0.0001	1.37	−0.3679	0.0383
Gestational age of delivery						
2‐Furanmethanol	Organoheterocyclic compounds	1.41	<0.0001	1.23	−0.4710	<0.000165
17a‐Hydroxypregnenolone	Lipids and lipid‐like molecules	1.40	<0.0001	1.53	−0.5138	<0.000126
(±)‐erythro‐Isoleucine	Organic acids and derivatives	1.26	<0.0001	1.30	−0.3541	0.0468
1‐Octene	Hydrocarbons	1.67	<0.0001	0.89	0.4769	<0.000158
Piperidine	Organoheterocyclic compounds	1.21	<0.0001	1.35	−0.4484	0.0101
L‐Carnitine	Organic nitrogen compounds	1.18	0.02	1.16	−0.4291	0.0143
Cadinene	Lipids and lipid‐like molecules	1.14	0.01	1.18	−0.4640	<0.000175
OKOHA‐PA	Lipids and lipid‐like molecules	1.04	<0.0001	1.55	−0.3888	0.0279

Abbreviations: FC, Fold change; UPLC‐HRMS, Ultra‐performance liquid chromatography‐high resolution mass spectrometry; VIP, variable influence on projection.

Thirdly, we analyzed sex‐specific differences in placental metabolomes. Interestingly, maternal GDM increased the differences in placental metabolites. Fourteen types of metabolomes showed significant changes between male and female offspring, in the GDM group (Table 3). In contrast, only two metabolites (4‐Dodecylphenol, 2S‐amino‐octadeca‐4E, 6E‐diene‐1,3R‐diol) showed sex‐specific differences in normal pregnant women. Most of the metabolites increased in females when compared with males. As an example, 5 kinds of LysoPE increased in females, including LysoPE (18:1(11Z)/0:0), LysoPE (0:0/18:1(11Z)), LysoPE (0:0/20:4(8Z,11Z,14Z,17Z)), LysoPE (0:0/20:4(5Z,8Z,11Z,14Z)), and LysoPE (20:4(5Z,8Z,11Z,14Z)/0:0). The levels of α‐linolenic acid were also slightly higher in females. Only four metabolites increased in male newborns, including MG (18:0/0:0/0:0), 10, 20‐dihydroxyeicosanoic acid, benzothiazole, and 1‐octene.

**TABLE 3:** Sex‐specific differences in placental metabolomes (10E8)

	Male	Female	*p*
GDM Group			
9‐Octadecenal	127.52 ± 38.18	158.12 ± 36.55	0.028
10,20‐Dihydroxyeicosanoic acid	75.99 ± 10.3	68.45 ± 8.68	0.034
Phytal	20.43 ± 7.57	26.38 ± 7.54	0.034
MG(18:0/0:0/0:0)	45.57 ± 5.53	41.08 ± 4.55	0.019
LysoPE(0:0/20:4(8Z,11Z,14Z,17Z))	15.94 ± 5.67	20.05 ± 4.67	0.034
LysoPE(0:0/20:4(5Z,8Z,11Z,14Z))	7.11 ± 1.09	9.78 ± 4.6	0.027
Benzothiazole	14.19 ± 1.34	12.98 ± 1.29	0.015
1‐Octene	12.02 ± 1.25	10.98 ± 1.22	0.024
PE(0:0/22:6(4Z,7Z,10Z,13Z,16Z,19Z))	3.2 ± 0.91	4.19 ± 1.65	0.041
LysoPE(20:4(5Z,8Z,11Z,14Z)/0:0)	2.8 ± 1.08	5.53 ± 4.94	0.034
α‐Linolenic Acid	2.16 ± 0.92	3.56 ± 2.32	0.029
LysoPE(0:0/18:1(11Z))	2.27 ± 0.96	3.31 ± 1.75	0.043
13‐Heptadecyn−1‐ol	1.45 ± 0.55	1.86 ± 0.53	0.043
LysoPE(18:1(11Z)/0:0)	2.16 ± 0.8	3.03 ± 0.91	0.017
Control Group			
4‐Dodecylphenol	3.04 ± 1.55	4.8±3.06	0.043
2S‐amino‐octadeca−4E,6E‐diene−1,3R‐diol	1.71 ± 1.16	3.04 ± 2.42	0.047

Abbreviation: GDM, Gestational diabetes mellitus.

## 4 DISCUSSION

GDM, the most common complication of pregnancy results from poorly controlled blood glucose and causes serious short‐term complications and long‐term consequences for mothers, fetuses, and newborns.^15^, ^16^ Insulin resistance, low‐grade inflammation, and endothelial cell dysfunction are considered its characteristic features. Increasing studies highlight the importance of metabolite alterations in GDM, and most efforts are focused on investigating biomarkers that could facilitate prediction and diagnosis.^17^, ^18^, ^19^ These studies suggested that changes in maternal metabolic status tend to affect the levels of various metabolites and metabolic homeostasis in the fetus and subsequently lead to an increased complication of pregnancy, such as macrosomia. However, the characteristics and molecular mechanism of metabolites in the maternal‐fetal microenvironment of GDM are still unclear. We described the placental metabolic profiling of GDM and preliminarily discussed the relationship between differentially expressed metabolites and clinical data obtained from mothers and newborns.

We confirmed that there were metabolic abnormalities in the placenta of GDM patients. Lipids and lipid‐like molecules were the main differential metabolites, especially changes in unsaturated fatty acids. Among 87 differentially expressed metabolites, lipids and lipid‐like molecules accounted for 62.1%, and significantly enriched the “biosynthesis of unsaturated fatty acids” and “fatty acid biosynthesis” pathways. The levels of related fatty acids increased significantly in the GDM placentas when compared to that of normal women. Linoleic acid and α‐linolenic acid may serve as good biomarkers for the prediction and diagnosis of GDM, but needs further study to confirm. Although the samples were different, the above results are consistent with other reports. Ogundipe,^20^ for example, reported that GDM women had elevated levels of n‐6 fats (linoleic acid, adrenic acid, palmitic acid), and depressed levels of n‐3 fats in erythrocytes. Zheng^21^ found that the levels of linolenic acid and arachidonic acid in maternal plasma were significantly elevated in GDM. In addition, Wu et al^22^ investigated the metabolomic of the umbilical cord blood in GDM and confirmed that GDM could induce unsaturated fatty acid metabolic abnormalities, and arachidonic acid (AA) might play an important role in the key metabolic pathway. These results will help guide the development of new potent biomarkers for GDM diagnosis and prevention and elucidate of the molecular mechanism of maternal‐fetal lipid transport. All fatty acids are expected to be part of the fatty acid synthesis pathway however it is not clear how this may be altered in GDM. However, whether they can be used as biomarkers for the prediction and diagnosis of GDM is not certain. DEMs as predictive biomarkers in late pregnancy is obviously not suitable, but they may provide us with information that can be used in future studies confirming similar results in maternal blood at earlier gestational stages.

Furthermore, we discussed the relationship between DEM and the clinical scenario. Several interesting results obtained are as follows: (1) Placental metabolites were related to the newborns birth weight. The level of PC (14:0/18:0) was especially negatively correlated with neonatal weight, which was similar to the results obtained by Alfano ^23^ and Lu.^24^ They demonstrated that three metabolites [PC (34:2), plasmalogen PC (36:4)/PC (O‐36:5), and a compound with m/z of 781.0545] were related to birth weight. Lu also reported that the levels of lysophosphatidylcholines (LPC) 14:0, 16:1, and 18:1 showed very strong and independent associations with birth weight. If these related metabolites in placenta or cord blood also have consistent availability in maternal blood, they could provide good indicators for noninvasive neonatal weight prediction. (2) Under GDM conditions, there is an increasing trend in sex‐specific differences in placental metabolomes. Among 87 DEM, 14 were significantly different in male and female offspring, with most of them increasing in female newborns. Some studies have also discussed this interesting topic. Saoi et al^25^ suggested that distinct placental metabolic profiling exists between male and female mice. Metabolites related to fatty acid oxidation and purine degradation were elevated in female. O'Neill^26^ profiled the metabolome of second trimester amniotic fluid (AF) from GDM women with a targeted metabolomics approach, and identified 44 and 58 metabolites in male and female offspring respectively. They confirmed that there were sex‐specific metabolic alterations in the GDM maternal‐fetal interface. At present, it is believed that the subtle sex‐specific differences in placental metabolism may reflect the most suitable conditions for fetal growth and development. Better understanding of these differences may aid timely intervention during pregnancy complications. (3) Surprisingly, there was no statistically significant correlation between the DEM and maternal BMI or blood glucose level. There were still some metabolic abnormalities in GDM even following strict control of blood glucose levels suggesting that mere control of glucose may not be enough to curtail GDM.

Nevertheless, the present preliminary study has some limitations. First, the results from this relatively small sample size need to be verified in an extended clinical study with an adequate sample size. Second, the objective of this metabolic study was limited to screening the molecular mechanism of maternal‐fetal energy transfer. The evaluation of clinical applications would be an additional important purpose. Because the placenta is not easy to sample during pregnancy and it can only be obtained after delivery, and it is obviously not possible to use our data, a clinical prediction. However, these DEMs in the placenta provide us with some clues and thoughts that can be used in future research to clarify their predictive and diagnostic value in maternal blood during earlier gestational weeks in patients evaluated for GDM. Third, we only sampled the placenta in the present study and did not include maternal blood or cord blood. It can therefore not fully reflect the full story of the metabolic regulation at the maternal‐fetal interface. On the other hand, because almost all of GDM can be effectively managed in the prenatal period, it is difficult to find pregnant women with GDM who have not received intervention, thereby making it difficult to obtain the original placental metabolic characteristics. In any case, the metabonomics of GDM is worthy of further study.

In conclusion, we described the placental metabolic profiling of GDM confirmed that lipids and lipid‐like molecules were the main differential metabolites, especially unsaturated fatty acids. Furthermore, even if maternal blood glucose level is well controlled, there are still metabolic abnormalities in GDM and sex‐specific alterations. Placental metabolites such as PC(14:0/18:0) correlate with the birth weight of newborns. These findings will contribute to reveal new molecular mechanisms of GDM.

## CONFLICT OF INTEREST

The authors declare that they have no competing interests.

## AUTHOR CONTRIBUTIONS

**Bin Yu** and **Huaiyan Wang** conceived the study and carried out the data assays. **Yuqi Yang**, **Fang Guo**, and **Wei Long** carried out laboratory tests and performed the statistical analysis. **Huaiyan Wang**, **Huihui Wang, and Zhaoping Pan** collected the clinical cases. **Yuqi Yang** and **Bin Yu** wrote the manuscript.

## ETHICAL APPROVAL

The study design and protocol were reviewed and approved by the ethics committee of Changzhou Maternal and Child Health Care Hospital affiliated with Nanjing Medical University (2021140). Consent has been obtained from each patient or subject after full explanation of the purpose and nature of all procedures used.

## Supporting information

Table S1

## Data Availability

The questionnaire and datasets used are available from the corresponding author on request.
