# Supplementary material for: Placental metabolic profiling in gestational diabetes mellitus: An important role of fatty acids
Source: J Clin Lab Anal. 2021 Nov 9;35(12):e24096. doi: 10.1002/jcla.24096 (PMC8649376; doi:10.1002/jcla.24096)
Supplement: Supplementary file 1 — Table S1 [file JCLA-35-e24096-s001.doc]

**Supplementary table 1. 87 differential metabolites (DEM) in this study**

| **Metabolites** | **Super Class** | **m/z** | **VIP** | **P-value** | **FC** |
| --- | --- | --- | --- | --- | --- |
| (±)-(Z)-2-(5-Tetradecenyl)cyclobutanone | Organic oxygen compounds | 282.279 | 22.31689856 | 8.16205E-06 | 1.308966 |
| 9,12,15-Octadecatrien-1-ol | Lipids and lipid-like molecules | 282.279 | 18.58818862 | 7.58992E-06 | 1.377081 |
| Farnesyl acetone | Unclassified | 280.263 | 15.21654353 | 8.99287E-06 | 1.451115 |
| 3,7,11,15-Tetramethyl-6,10,14-hexadecatrien-1-ol | Lipids and lipid-like molecules | 310.31 | 11.34240755 | 1.49171E-05 | 1.45726 |
| Hexadecenal | Lipids and lipid-like molecules | 256.263 | 9.90113852 | 3.74119E-05 | 1.388981 |
| Oleamide | Lipids and lipid-like molecules | 280.265 | 9.655945341 | 0.001445796 | 1.279707 |
| 9-Octadecenal | Lipids and lipid-like molecules | 284.295 | 8.908166384 | 1.57087E-05 | 1.386157 |
| Palmitic acid | Lipids and lipid-like molecules | 255.233 | 6.729531594 | 0.014847754 | 1.225984 |
| Linoleamide | Lipids and lipid-like molecules | 278.249 | 6.510614776 | 0.000468444 | 1.457144 |
| Arachidonic acid | Lipids and lipid-like molecules | 303.233 | 6.321232179 | 0.020241586 | 1.272687 |
| Geranylcitronellol | Lipids and lipid-like molecules | 310.31 | 6.197328543 | 2.83191E-05 | 1.497833 |
| Stearoylethanolamide | Organic nitrogen compounds | 308.296 | 5.827751743 | 0.0019221 | 1.326587 |
| Oleic Acid | Lipids and lipid-like molecules | 281.249 | 5.195646373 | 0.020584658 | 1.297575 |
| PC(14:0/18:0) | Lipids and lipid-like molecules | 734.568 | 5.115321136 | 0.011807809 | 0.808477 |
| Linoleic acid | Lipids and lipid-like molecules | 279.233 | 5.065196437 | 0.007795339 | 1.269046 |
| Stearic acid | Lipids and lipid-like molecules | 283.264 | 4.868034441 | 0.02183875 | 1.185825 |
| 4-(1,1,3,3-Tetramethylbutyl)-phenol | Benzenoids | 205.16 | 4.805916849 | 0.000191697 | 0.895246 |
| 10,20-Dihydroxyeicosanoic acid | Lipids and lipid-like molecules | 389.29 | 4.62126068 | 1.28353E-05 | 0.868129 |
| Piriprost | Unclassified | 434.229 | 4.131457483 | 0.005545581 | 0.868143 |
| Phytal | Lipids and lipid-like molecules | 312.325 | 3.764843881 | 3.24749E-05 | 1.478139 |
| MG(18:0/0:0/0:0) | Lipids and lipid-like molecules | 403.306 | 3.622384515 | 5.72567E-06 | 0.87024 |
| 3-(5,6,6-Trimethylbicyclo[2.2.1]hept-1-yl)cyclohexanol | Lipids and lipid-like molecules | 254.247 | 3.475213566 | 0.000232662 | 1.520803 |
| Ethyl isopropyl disulfide | Organosulfur compounds | 137.046 | 3.3467264 | 0.000649612 | 1.210741 |
| Sphinganine | Organic nitrogen compounds | 282.28 | 3.258891697 | 0.000651984 | 1.425411 |
| Palmitic amide | Lipids and lipid-like molecules | 254.249 | 2.916536598 | 0.000731826 | 1.478646 |
| 4,8,12-trimethyl-tridecanoic acid | Lipids and lipid-like molecules | 274.274 | 2.878797838 | 3.47708E-05 | 1.471039 |
| 1-Monopalmitin | Lipids and lipid-like molecules | 375.274 | 2.595269164 | 5.25929E-05 | 0.864791 |
| Desmethylmianserin | Unclassified | 251.155 | 2.543839067 | 4.73151E-08 | 1.175185 |
| Oxypurinol | Organoheterocyclic compounds | 153.041 | 2.480391267 | 0.000114354 | 1.437552 |
| LysoPE(0:0/20:4(8Z,11Z,14Z,17Z)) | Lipids and lipid-like molecules | 502.292 | 2.462525871 | 0.001861703 | 1.28743 |
| 4-Dodecylphenol | Unclassified | 280.263 | 2.416329409 | 0.000247426 | 1.981252 |
| 1-Phenyl-1,2-propanedione | Benzenoids | 166.086 | 2.380692637 | 0.000244839 | 1.37374 |
| LysoPE(0:0/20:4(5Z,8Z,11Z,14Z)) | Lipids and lipid-like molecules | 500.279 | 2.290584634 | 7.23126E-05 | 1.511412 |
| Imidazolepropionic acid | Organoheterocyclic compounds | 123.056 | 2.272012397 | 3.61409E-07 | 1.664284 |
| 2-(5,8-Tetradecadienyl)cyclobutanone | Organooxygen compounds | 280.263 | 2.260333351 | 0.000474914 | 1.874892 |
| aminocaproic acid | Lipids and lipid-like molecules | 132.102 | 2.245890588 | 0.000376327 | 1.319087 |
| Ambronide | Organoheterocyclic compounds | 254.247 | 2.218383653 | 7.73036E-06 | 1.458052 |
| 6-[1]-ladderane hexanol | Lipids and lipid-like molecules | 278.248 | 2.149472693 | 6.98982E-05 | 1.518087 |
| Phenylpyruvic acid | Benzenoids | 182.081 | 2.039912656 | 0.000105976 | 1.365187 |
| 2S-amino-octadeca-4E,6E-diene-1,3R-diol | Unclassified | 296.259 | 2.018283559 | 0.00046947 | 2.185252 |
| 6-Acetyl-2,2-dimethyl-2H-1-benzopyran | Organoheterocyclic compounds | 183.082 | 2.008070508 | 6.88236E-12 | 1.247061 |
| N-tetradecanoyl-L-Homoserine lactone | Unclassified | 292.228 | 1.738890993 | 0.000546193 | 1.440643 |
| Benzothiazole | Organoheterocyclic compounds | 136.022 | 1.726113953 | 2.54894E-05 | 0.897512 |
| PC(16:0/0:0) | Lipids and lipid-like molecules | 496.339 | 1.720846032 | 0.007275263 | 1.395489 |
| 1-Octene | Hydrocarbons | 130.159 | 1.671655227 | 1.97324E-05 | 0.890942 |
| LysoPA(0:0/16:0) | Lipids and lipid-like molecules | 409.236 | 1.595759138 | 0.030576647 | 1.252216 |
| 2-tetradecenal | Lipids and lipid-like molecules | 228.232 | 1.580734925 | 0.000698551 | 1.454637 |
| Porson | Phenylpropanoids and polyketides | 387.18 | 1.570345053 | 0.008338232 | 1.140772 |
| Linalyl propionate | Lipids and lipid-like molecules | 228.196 | 1.529477223 | 1.51553E-05 | 0.9038 |
| PE(0:0/22:6(4Z,7Z,10Z,13Z,16Z,19Z)) | Lipids and lipid-like molecules | 524.278 | 1.522992048 | 5.60818E-05 | 1.524912 |
| LysoPE(20:4(5Z,8Z,11Z,14Z)/0:0) | Lipids and lipid-like molecules | 500.278 | 1.502746255 | 0.021190486 | 1.757021 |
| PE(22:6(4Z,7Z,10Z,13Z,16Z,19Z)/0:0) | Lipids and lipid-like molecules | 526.293 | 1.497255516 | 0.004522562 | 1.324594 |
| N-tetradecanoyl-homoserine lactone | Lipids and lipid-like molecules | 292.228 | 1.473874527 | 0.001862245 | 1.532303 |
| Palmitoleamide | Lipids and lipid-like molecules | 252.233 | 1.464521833 | 0.000953099 | 1.65911 |
| Xanthine | Organoheterocyclic compounds | 151.026 | 1.458067213 | 0.046890678 | 1.133715 |
| Daimuron | Unclassified | 249.141 | 1.452411349 | 6.67131E-09 | 1.227377 |
| 2-Furanmethanol | Organoheterocyclic compounds | 116.071 | 1.414598361 | 0.001316069 | 1.231569 |
| α-Linolenic Acid | Lipids and lipid-like molecules | 296.258 | 1.41138967 | 0.000604225 | 1.841597 |
| 17a-Hydroxypregnenolone | Lipids and lipid-like molecules | 315.232 | 1.404694837 | 0.00043249 | 1.529225 |
| SALSOLINE | Organoheterocyclic compounds | 194.118 | 1.348890657 | 1.86653E-05 | 0.904022 |
| Margaric acid | Lipids and lipid-like molecules | 269.249 | 1.339613504 | 0.001807621 | 1.334684 |
| Phthalic acid | Benzenoids | 149.023 | 1.338431924 | 0.000151251 | 0.909577 |
| 8,11-Heptadecadienal | Lipids and lipid-like molecules | 268.263 | 1.321893928 | 0.000367335 | 1.632613 |
| (±)-erythro-Isoleucine | Amino acid | 132.102 | 1.26058821 | 0.000812538 | 1.296499 |
| MG(0:0/i-16:0/0:0) | Lipids and lipid-like molecules | 659.548 | 1.243200325 | 6.25805E-06 | 0.830469 |
| (E)-2-Octene | Hydrocarbons | 130.159 | 1.231454373 | 0.000125864 | 0.9091 |
| 6-[3]-ladderane-1-hexanol | Lipids and lipid-like molecules | 280.263 | 1.217276416 | 0.001512348 | 1.852046 |
| Piperidine | Organoheterocyclic compounds | 130.087 | 1.211394388 | 0.000215302 | 1.345207 |
| L-Carnitine | Organic nitrogen compounds | 162.112 | 1.18383105 | 0.023002834 | 1.156529 |
| 8E-Heneicosene | Lipids and lipid-like molecules | 339.327 | 1.166982372 | 0.046068996 | 1.293401 |
| (3S,6E,10E)-1,6,10,14-Phytatetraen-3-ol | Lipids and lipid-like molecules | 308.294 | 1.163021711 | 4.04077E-05 | 1.56939 |
| Cadinene | Lipids and lipid-like molecules | 253.217 | 1.141249068 | 0.011837186 | 1.175622 |
| Palmitoleoyl Ethanolamide | Organic nitrogen compounds | 320.256 | 1.118918246 | 0.000381126 | 1.932215 |
| LysoPE(0:0/18:1(11Z)) | Lipids and lipid-like molecules | 478.294 | 1.11356232 | 0.004969217 | 1.44371 |
| PS(O-18:0/0:0) | Lipids and lipid-like molecules | 556.326 | 1.112972602 | 0.034806767 | 1.237998 |
| 2,6-Nonadien-1-ol | Lipids and lipid-like molecules | 158.154 | 1.111839181 | 1.77456E-05 | 0.886028 |
| (4E,8E,10E-d18:3)sphingosine | Lipids and lipid-like molecules | 294.244 | 1.1018679 | 0.001200903 | 2.626252 |
| LysoPC(15:0) | Lipids and lipid-like molecules | 480.31 | 1.100859447 | 0.034540468 | 1.209077 |
| 5-amino-pentanoic acid | Lipids and lipid-like molecules | 118.087 | 1.09800065 | 0.002483618 | 1.211024 |
| MG(a-21:0/0:0/0:0)[rac] | Lipids and lipid-like molecules | 445.354 | 1.070370402 | 0.000265974 | 0.771395 |
| 2,4,6-Octatriyn-1-ol | Lipids and lipid-like molecules | 136.076 | 1.055526575 | 6.06223E-05 | 1.400194 |
| Armillarin | Lipids and lipid-like molecules | 415.211 | 1.046165417 | 0.03792698 | 1.14333 |
| OKOHA-PA | Lipids and lipid-like molecules | 619.289 | 1.039652366 | 0.00122184 | 1.5499 |
| PC(6:0/6:0)[S] | Unclassified | 498.25 | 1.033887141 | 0.000449066 | 2.39351 |
| 2-Hexylidenecyclopentanone | Organooxygen compounds | 184.17 | 1.026970647 | 1.40011E-05 | 1.442598 |
| 13-Heptadecyn-1-ol | Lipids and lipid-like molecules | 270.279 | 1.011576182 | 3.98657E-05 | 1.478275 |
| LysoPE(18:1(11Z)/0:0) | Lipids and lipid-like molecules | 480.308 | 1.010391506 | 0.001964725 | 1.347353 |
